# A theoretical calculation method of seismic shear key pounding based on continuum model

**DOI:** 10.1016/j.mex.2023.102370

**Published:** 2023-09-07

**Authors:** Chen Shutong, Fadzli Mohamed Nazri, An Wenjun, Fu Hao

**Affiliations:** a School of Civil Engineering, Engineering Campus, Universiti Sains Malaysia, Nibong Tebal, Penang 14300, Malaysia; b School of Civil Engineering, Jiangxi University of Engineering, Fairy Lake Campus, Yushui District, Xinyu 338000, China; c Faculty of Civil Engineering, Universiti Teknologi Malaysia UTM, Skudai, Johor 81310, Malaysia

**Keywords:** Transient wave function expansion method, Mode superposition method, Combined transient internal force method, Duhamel integration method, Theoretical Calculation of horizontal Pounding Calculation of Shear Key Based on Continuum Model

## Abstract

The evolution of shear key design for bridges is accompanied by research on structural earthquake resistance. However, the vast majority of pounding forces, responses, and corresponding data for the study and design of shear keys have been based on expensive experimentalism and imprecise empiricism approaches for decades. Hence, strengthening theoretical study on seismic performance of shear key is essential. In this paper, a “Beam-Spring-Beam + Concentrated Mass” continuum dynamic model is proposed. Meanwhile, the transient wave function expansion method and the mode superposition method are applied to determine the analytical expression of the dynamic response from the girder and pier system (pier and cap beam). Furthermore, the combined transient internal force method and Duhamel integration method are introduced to assess the elastic pounding process. Through programming and numerical analysis, a series of pounding response data related to the shear key under various working circumstances will be explored. As mentioned above, the proposed theoretical method can optimize shear key design and boost the reliability of seismic limiting devices in the future.

• Establishing a feasible “Beam-Spring-Beam + Concentrated Mass” continuum model of girders and piers based on a two-span continuous girder bridge.
• Deriving the analytical solutions of responses by conducting the response equations under horizontal seismic excitation (containing orthonormality verification).
• Simulating the pounding process by embedding elastic pounding calculation methods into Continuum Model.

## Graphical abstract

**Figure:**
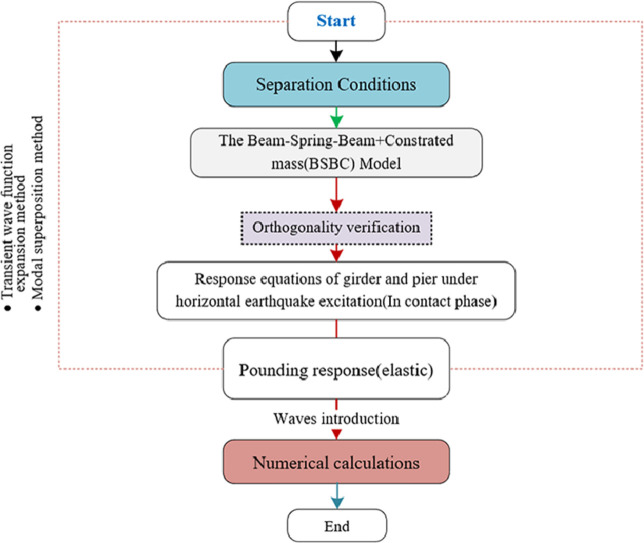

Specifications table

**Table:** 

Subject area:	Engineering
More specific subject area:	*Structural and Earthquake Engineering*
Name of your method:	*Theoretical Calculation of horizontal Pounding Calculation of Shear Key Based on Continuum Model*
Name and reference of original method:	*A.K.Chopra. Dynamics of Structures, Prentice Hall: Upper Saddle River.1995.*
Resource availability:	*Not Applicable*

**Method details**

## Introduction

**Fig. 1:**
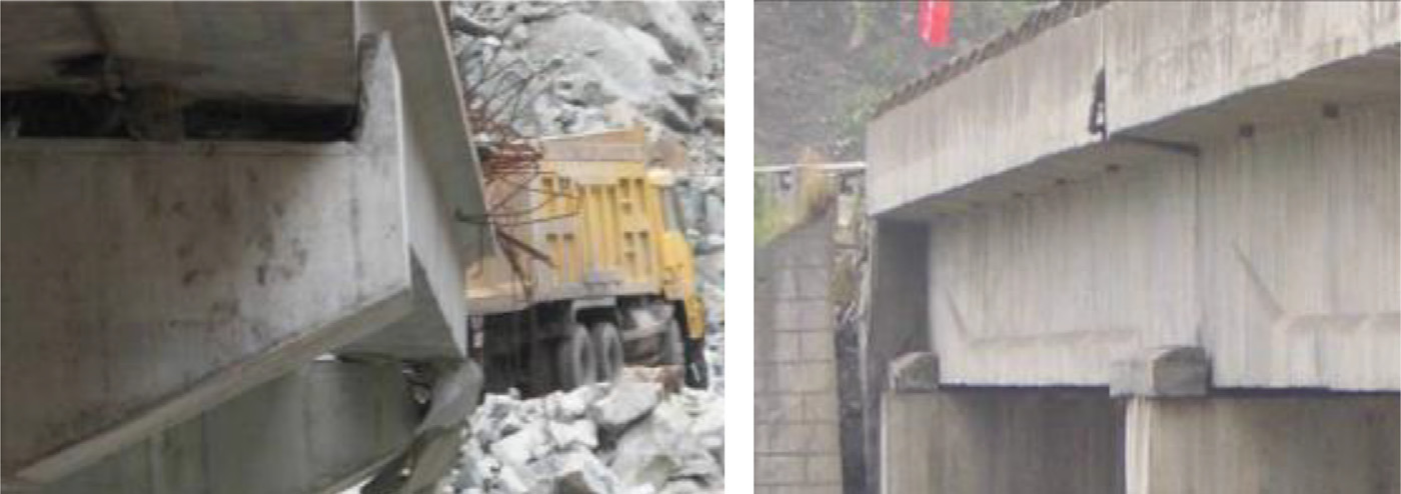
The observed destruction of shear keys under Wenchuan earthquake in 2008 [3].

Shear key pounding is the most common form of damage caused by seismic events to bridge structures. For instance, during the 1994 Northridge earthquake, Shear key poundings were found in large numbers within the bridge structure. During the Chichi earthquake that occurred in 1999, the Tong-feng bridge experienced a significant transverse slide, with the girder even sliding off the bearings [1]. During the Wenchuan earthquake in 2008, The rate of shear key failure occurring to girder-bridge structures reached 16.8%, while the pier failure rate was merely 2.4% [2]. As indicated by the momentous damage observed in various earthquakes, shear keys play a pivotal role as the anti-seismic mechanism for the bridge system. Therefore, it is imperative to fully understand their pounding theory and process (Fig. 1).

Currently, Methods for structural seismic analysis were mainly based on finite element software and experiment manners, for instance, Ozturk Analysed seismic behavior of two monumental buildings in Cappadocia region of Turkey and systematically verified the impact of walls on structural seismic resistance [4]. Kim et al. investigated the behavior of reinforced concrete (RC) piers under horizontal and vertical earthquake excitation by experiments [5].

This study aims to establish a novel theoretical framework for the horizontal pounding analysis of girder-shear key based on continuum response models [6,7]. By employing the proposed model, it is possible to analyze the compromised interaction of bridge structures in detail both accurately and flexibly, including but not limited to simulating the process of horizontal and vertical response from the bridge structures. Meanwhile, it is applicable in practice considering the complex boundary conditions of structures in the presence of seismic action. As revealed by extensive research, similar continuum models can be used to enable the high exactitude and excellent fitting precision required for the dynamic response of bridge structures. For example, Clvalek et al. proposed a discrete singular convolution (DSC) and harmonic differential quadrature (HDQ) theoretical methods to solve the geometrically non-linear dynamic problem for rectangular plates resting on elastic foundation, and the numerical solution appeared that the shear parameter G of the Pasternak foundation and stiffness parameter K of the Winkler foundation have been considered to have an enormous influence on the dynamic response of the plates [8]. Atdin et al. developed a new approach to optimize the design of cantilever beams under different harmonic virbrations based on a Timoshenko beam model and elastic springs [9]. In this way, the dynamic behavior of the bridge can be accurately simulated and addressed in the context of seismic action [10], [11], [12], [13], [14], [15]. Therefore, the application of this model is beneficial in developing theoretical guidance for the design of seismic shear key in this field, which contributes a novel solution to the research and design of anti-seismic device for bridges. Thus, the current reliance solely on experiments and empirical studies can be addressed.

## Establishment of model

**Fig. 2:**
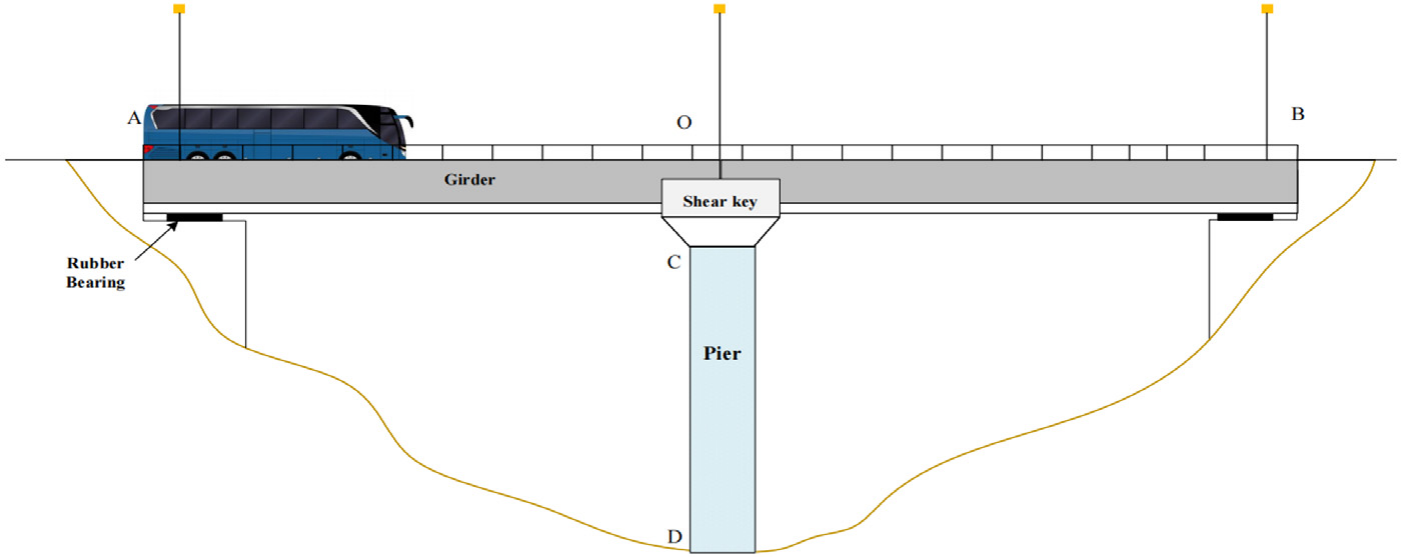
Simplified diagram of the double span simply supported girder bridge.

Currently, girder bridge stand in dominant position among all bridge structures (e.g., this structure accounts for 74% of all highway bridges in China). Thereby, this study employs a “double span simply supported girder bridge” and establishes a “Beam-Spring-Beam + Concentrated Mass” continuum model to imitate the seismic response behavior of shear key pounding (Fig. 2). Meanwhile, the participation of transient wave function expansion method and mode superposition method afforded substantial assistance in deriving the vertical displacement and horizontal pounding responses of bridge structures. Additionally, this study also proposes a demonstration method for the verification of orthogonality of transient wave functions of bridge structures under horizontal seismic action.

**Fig. 3:**
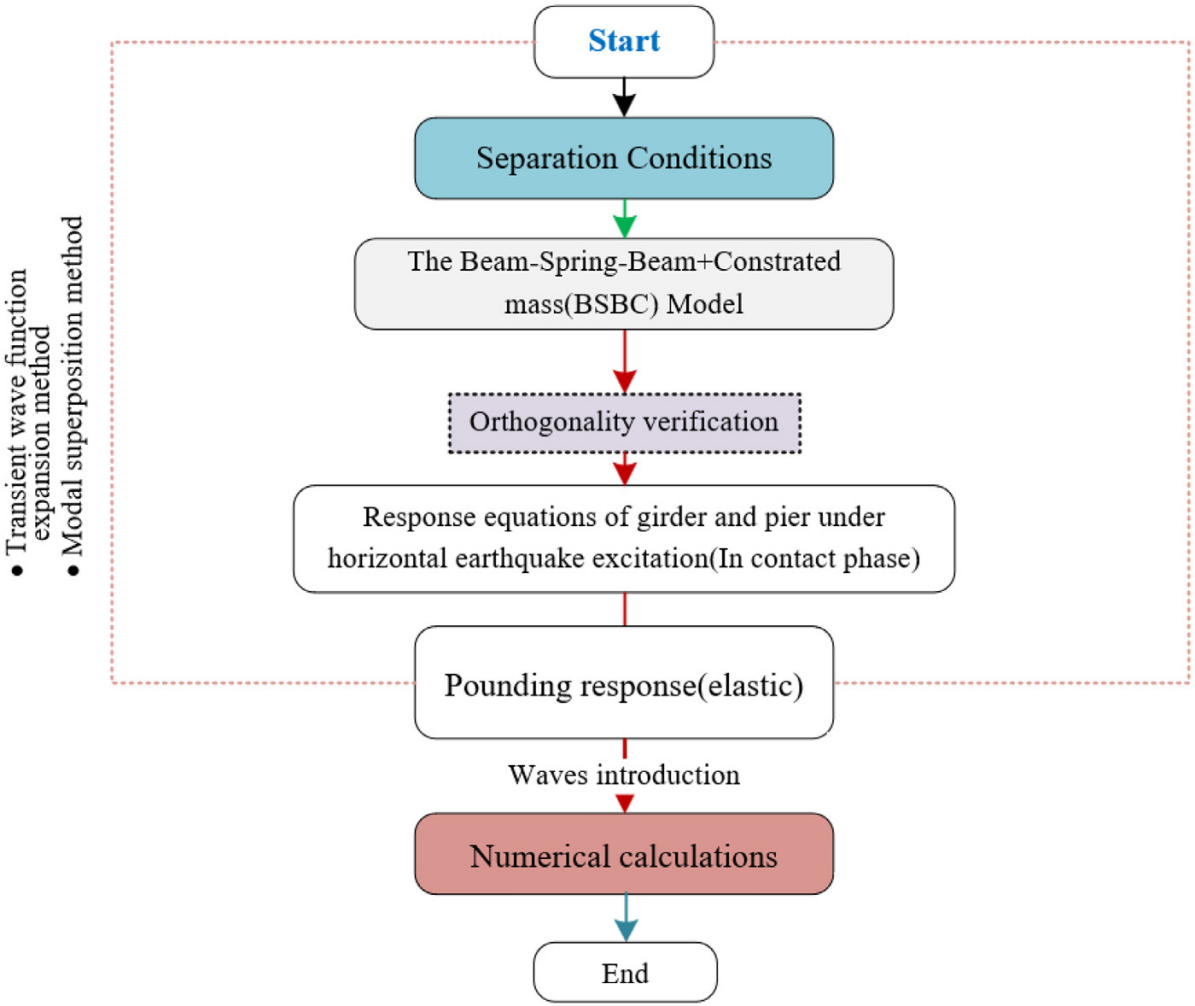
Flowchart of theoretical methodology.

The specific process is as follows: (Fig. 3).

### Horizontal seismic response of the girder and pier systems

To study the dynamic response of the bridge and pier system (pier and cap beam) in this state, a simplified model is introduced and explained as follows:

(i) A “Beam-Spring-Beam + Concentrated mass” model was established under horizontal seismic action. The piers are simplified as equal cross-section Bernoulli-Euler beams, while the  cap beam (containing shear key) is equivalently represented as a concentrated mass Mk. The shear key has a shear stiffness of Kv and is rigidly connected to the top of the pier. Assuming that there are no horizontal interaction forces between the girder and shear key at the initial moment.
(ii) The span length of the girders is denoted as x_0_, with a cross-sectional area of *A_b_*. The density of the reinforced concrete material is represented as *ρ_b_*, and the elastic modulus is denoted as *E_b_*. The moment of inertia of the cross-section is denoted as *I_b_*. The height of pier CD is denoted as *H*, with a cross-sectional area of *A_c_*. The material density of the pier is denoted as *ρ_c_*, and the elastic modulus is represented as *E_c_*. The moment of inertia of the cross-section is denoted as *I_c_*.
(iii) The horizontal seismic excitation is represented by *D(t)*, and it is synchronously inputted from the bottom of the piers and the girder supports. The seismic traveling wave effect and the coupling effect of vertical and horizontal seismic actions are not under consideration in the analysis of horizontal seismic action.
(iv) The girders and pier system are assumed to remain in an elastic state, disregarding plastic deformation of the structure. Due to the minimal damping in concrete materials (typically around 5%), the damping effect is neglected in the structural vibration analysis.
(v) The combined transient internal force method is implanted in pounding process, which is equivalent to pounding process as the difference in displacement between transient responses of the bridge structure. The magnitude of the collision force is determined by the compression of the spring. This method effectively circumvents the need to solve nonlinear equations and mitigates convergence issues in numerical computations, while yielding satisfactory fitting results.
(vi) The influence of horizontal seismic action on the vertical height of the piers is disregarded. Meanwhile, the friction on the contact surface of the bearing.

**Fig. 4:**
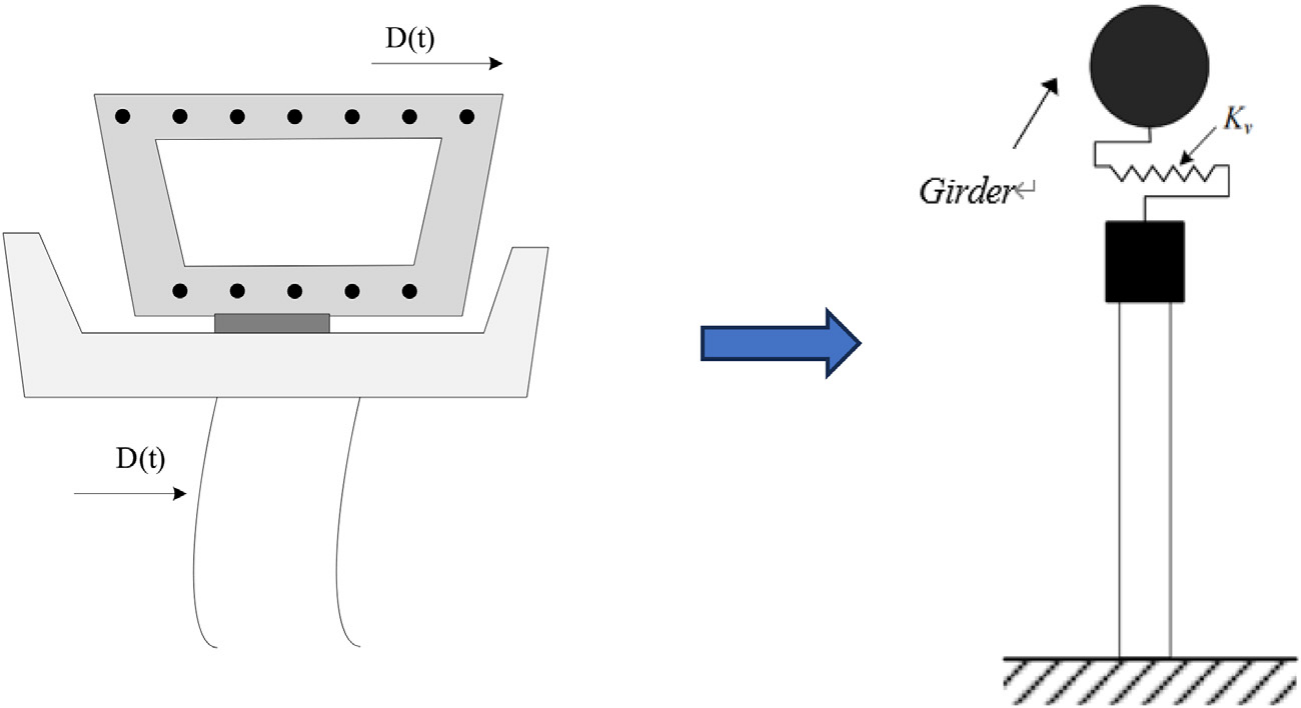
Pounding process and pier system as a beam+ concentrated model (cross-section view).

**Fig. 5:**
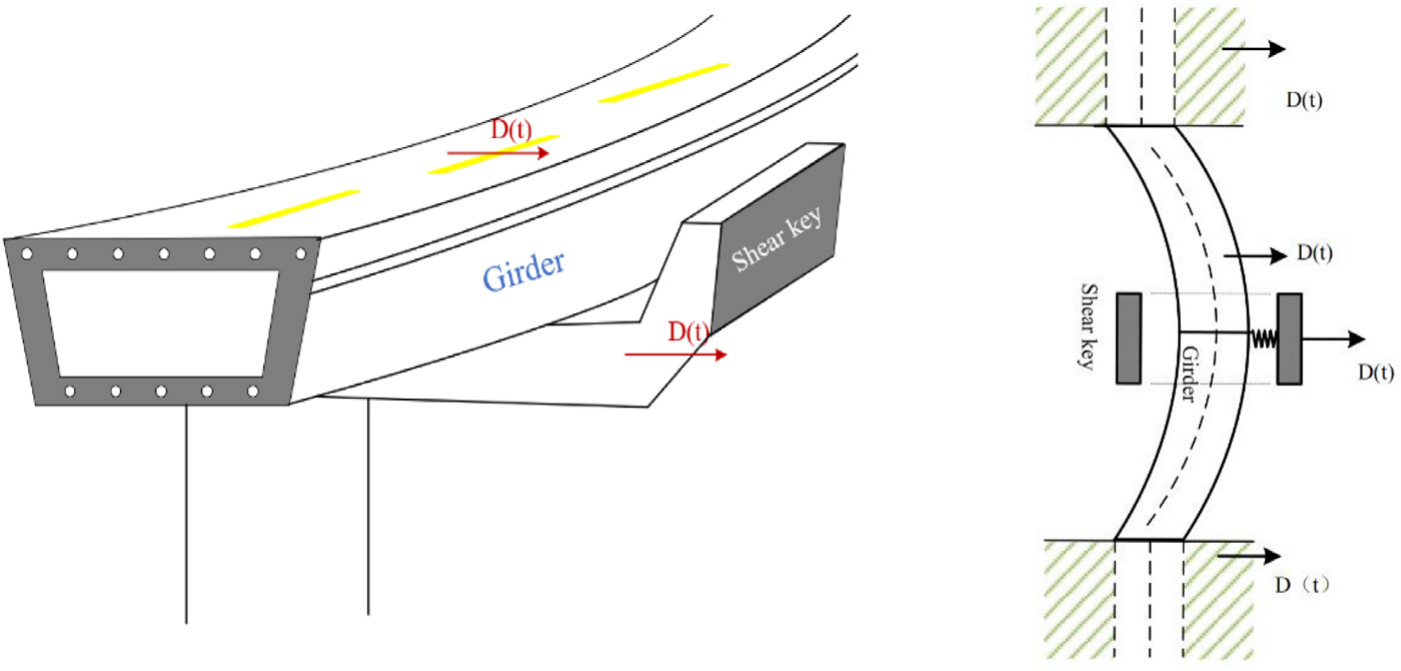
Pounding process and girders as a continuous beam.

Figs. 4 and 5 illustrate the simplified pounding process and response.

### Establishment of horizontal displacement equations

As known, the girder and shear key are prone to experiencing repeated poundings under horizontal seismic excitation. To facilitate the calculation of this process, the total horizontal displacement is expressed by *X*_1_(*x*), *X*_2_(*x*), *X_c_*(*ξ*). These displacements comprise the static horizontal displacements *X*_1_*_s_*(*x*), *X*_2_*_s_*(*x*), *X_cs_*(*ξ*), the rigid body displacements introduced by *X*_1g_(*x*), *X*_2_*_g_*(*x*), *X_cg_*(*ξ*), and the dynamic displacements is denoted as *X*_1_*_d_*(*x*), *X*_2_*_d_*(*x*), *X_cd_*(*ξ*), The natural frequency of the structure is$\;\mu_{i}$, and their satisfaction can be expressed through the following Eq. (1):

(1) $$\begin{array}{ccc} & & X_{1}\left(x,t\right)=X_{1s}\left(x\right)+X_{1g}\left(x,t\right)+X_{1d}\left(x,t\right)\\ & & X_{2}\left(x,t\right)=X_{2s}\left(x\right)+X_{2g}\left(x,t\right)+X_{2d}\left(x,t\right)\\ & & X_{c}\left(\xi ,t\right)=X_{cs}\left(\xi \right)+X_{cg}\left(\xi ,t\right)+X_{cd}\left(\xi ,t\right) \end{array}$$

In Eq. (1), the static horizontal displacements as motioned in Eq. (2):

(2) $$X_{1s}\left(x\right)=X_{2s}\left(x\right)=X_{cs}\left(\xi \right)=0$$

And the magnitudes of rigid body displacement equivalent to horizontal earthquake excitation. From the simultaneity of incentive effects, it can be expressed as follows Condition (3):

(3) $$X_{1g}\left(x,t\right)=X_{2g}\left(x,t\right)=X_{cg}\left(\xi ,t\right)=D\left(t\right)$$

Under horizontal earthquake excitation, the total wave equation of girders OA,OB and pier CD can be expressed as following Eq. (4):

(4) $$\begin{array}{ccc} & & OA:E_{b}I_{b}\frac{\partial^{4}X_{1}\left(x,t\right)}{\partial x^{4}}+\rho_{b}A_{b}\frac{\partial^{2}X_{1}\left(x,t\right)}{\partial t^{2}}=0\\ & & OB:E_{b}I_{b}\frac{\partial^{4}X_{2}\left(x,t\right)}{\partial x^{4}}+\rho_{b}A_{b}\frac{\partial^{2}X_{2}\left(x,t\right)}{\partial t^{2}}=0\\ & & CD:\frac{E_{c}I_{c}\partial^{4}X_{c}\left(\xi ,t\right)}{\partial \xi^{4}}+\rho_{c}A_{c}\frac{\partial^{2}X_{c}\left(\xi ,t\right)}{\partial t^{2}}=0 \end{array}$$

Based on substituting the given expressions (1)–(3) into (4), the horizontal dynamic displacement of OA,OB and CD can be converted into the following Eq. (5):

(5) $$\begin{array}{ccc} & & OA:E_{b}I_{b}\frac{\partial^{4}X_{1d}\left(x,t\right)}{\partial x^{4}}+\rho_{b}A_{b}\frac{\partial^{2}X_{1d}\left(x,t\right)}{\partial t^{2}}=-\rho_{b}A_{b}\frac{\partial^{2}X_{1g}\left(x,t\right)}{\partial t^{2}}\\ & & OB:E_{b}I_{b}\frac{\partial^{4}X_{2d}\left(x,t\right)}{\partial x^{4}}+\rho_{b}A_{b}\frac{\partial^{2}X_{2d}\left(x,t\right)}{\partial t^{2}}=-\rho_{b}A_{b}\frac{\partial^{2}X_{2g}\left(x,t\right)}{\partial t^{2}}\\ & & CD:\frac{E_{c}I_{c}\partial^{4}X_{cd}\left(\xi ,t\right)}{\partial \xi^{4}}+\rho_{c}A_{c}\frac{\partial^{2}X_{cd}\left(\xi ,t\right)}{\partial t^{2}}=-\rho_{c}A_{c}\frac{\partial^{2}X_{cg}\left(\xi ,t\right)}{\partial t^{2}} \end{array}$$

Simultaneously, the homogeneous equation of Eq. (5) can be represented as follows (6):

(6) $$\begin{array}{ccc} & & OA:a_{1}^{2}\frac{\partial^{4}X_{1d}\left(x,t\right)}{\partial x^{4}}+\frac{\partial^{2}X_{1d}\left(x,t\right)}{\partial t^{2}}=0\\ & & OB:a_{1}^{2}\frac{\partial^{4}X_{2d}\left(x,t\right)}{\partial x^{4}}+\frac{\partial^{2}X_{2d}\left(x,t\right)}{\partial t^{2}}=0\\ & & CD:{\bar{c}}_{1}^{2}\frac{\partial^{4}X_{cd}\left(\xi ,t\right)}{\partial \xi^{4}}+\frac{\partial^{2}X_{cd}\left(\xi ,t\right)}{\partial t^{2}}=0 \end{array}$$

where a_1_, c_1_ are the horizontal wave number of girder and pier and can be inferred as: $a_{1}=\sqrt{\frac{E_{b}I_{b}}{\rho_{b}A_{b}}},{\bar{c}}_{1}=\sqrt{\frac{E_{c}I_{c}}{\rho_{c}A_{c}}}$.

### Verification of orthogonality for the mode shapes of the girders and pier system

The premise of solving the characteristic function coefficient of the modal function and modal mass is to construct and prove its orthogonality. According to the transient wave function expansion method, the dynamic displacement term of the bridge structures(OA,OB,CD)can be expanded as follows:

(7) $$\begin{array}{ccc} & & X_{1d}\left(x,t\right)=\sum_{i=1}^{\infty }\psi_{i1}\left(x\right)T_{i}\left(t\right),\\ & & X_{2d}\left(x,t\right)=\sum_{i=1}^{\infty }\psi_{i2}\left(x\right)T_{i}\left(t\right)\\ & & X_{cd}\left(\xi ,t\right)=\sum_{i=1}^{\infty }\psi_{ic}\left(\xi \right)T_{i}\left(t\right) \end{array}$$

where $\psi_{i1}\left(x\right),\psi_{i2}\left(x\right),\psi_{ic}\left(\xi \right)$ are the wave mode (model) functions of the girder and pier, and $T_{i}\left(t\right)$ denotes the time function of waves in the bridge structures.

The orthogonality verification in this section is significantly different from the rod and beam components, it requires considering more complicated situations in which the shear key as a concentrated mass enormously participated in horizontal vibrations. If adopting boundary conditions to solve the problem consistent with the boundary conditions, which require an enormous workload and are difficult to replicate.

The functionals and calculus of variations based on energy conservation are considered ideal methods that can immensely optimize the solution process and make it more applicable. Then, attempting to employ this method to derive the orthogonality of the structure.

For the girder and pier system, the maximum elastic potential energy of its horizontal vibration is as follows:

(8) $$E_{Q,\max}=\frac{1}{2}E_{b}I_{b}\left[\int_{-x_{0}}^{0}{\psi^{\prime \prime }}_{i1}^{2}\left(x\right)dx+\int_{0}^{x_{0}}{\psi^{\prime \prime }}_{i2}^{2}\left(x\right)dx+\int_{0}^{H}{\psi^{\prime \prime }}_{ic}^{2}\left(\xi \right)d\xi \right]$$

And the maximum kinetic energy can be expressed as:

(9) $$E_{K,\max}=\frac{1}{2}\mu_{i}^{2}\rho_{b}A_{b}\left[\int_{-x_{0}}^{0}\psi_{i1}^{2}\left(x\right)dx+\int_{0}^{x_{0}}\psi_{i2}^{2}\left(x\right)dx\right]+\frac{1}{2}\mu_{i}^{2}\rho_{c}A_{c}\int_{0}^{H}\rho_{c}A_{c}\varphi_{ic}^{2}\left(\xi \right)d\xi +\frac{1}{2}\mu_{i}^{2}M_{k}\psi_{ic}^{2}\left(H\right)$$

Introducing $\varphi_{jc}$ and redefine $\varphi_{c}\left(\xi \right)$ as:

(10) $$\begin{array}{ccc} & & \psi_{i1}\left(x\right)=a_{i}\psi_{i1}\left(x\right)+a_{j}\psi_{i1}\left(x\right)\\ & & \psi_{i2}\left(x\right)=a_{i}\psi_{i2}\left(x\right)+a_{j}\psi_{i2}\left(x\right)\\ & & \psi_{ic}\left(\xi \right)=a_{i}\psi_{ic}\left(\xi \right)+a_{j}\psi_{jc}\left(\xi \right) \end{array}$$

Rewrite Eqs. (8) and (9) as:

(11) $$\begin{array}{ccc} E_{Q,\max} & = & \frac{1}{2}(a_{i}^{2}K_{ii1}+2a_{i}a_{j}K_{ij1}+a_{j}^{2}K_{jj1}]+\frac{1}{2}\left(a_{i}^{2}K_{ii2}+2a_{i}a_{j}K_{ij2}+a_{j}^{2}K_{jj2}\right)\\ & & +\;\frac{1}{2}\left(a_{i}^{2}K_{iic}+2a_{i}a_{j}K_{ijc}+a_{j}^{2}K_{jjc}\right) \end{array}$$

(12) $$\begin{array}{ccc} E_{K\max} & = & \frac{1}{2}\mu_{i}^{2}(a_{i}^{2}M_{ii1}+2a_{i}a_{j}M_{ij1}+a_{j}^{2}M_{jj1}]+\frac{1}{2}\mu_{i}^{2}(a_{i}^{2}M_{ii2}+a_{i}a_{j}M_{ij2}+a_{j}^{2}M_{jj2}]\\ & & +\;\frac{1}{2}\mu_{i}^{2}\left(a_{i}^{2}M_{iic}+a_{i}a_{j}M_{ijc}+a_{j}a_{i}M_{jic}+a_{j}^{2}M_{jjc}\right) \end{array}$$

Where *a_i_, a_j_* are the correlation coefficients.

(13) $$\begin{array}{ccc} & & K_{rs1}=E_{b}I_{b}\int_{-x_{o}}^{0}{\psi^{\prime \prime }}_{r1}\left(x\right){\psi^{\prime \prime }}_{s1}\left(x\right)dxM_{rs1}=\rho_{b}A_{b}\int_{-x_{o}}^{0}\psi_{r1}\left(x\right)\psi_{s1}\left(x\right)dx*r,s=i,j\\ & & K_{rs2}=E_{b}I_{b}\int_{0}^{x_{0}}{\psi^{\prime \prime }}_{r2}\left(x\right){\psi^{\prime \prime }}_{s2}\left(x\right)dxK_{rs2}=\rho_{b}A_{b}\int_{0}^{x_{0}}\psi_{r2}\left(x\right)\psi_{s2}\left(x\right)dx\\ & & K_{rsc}=E_{c}I_{c}\int_{0}^{H}{\psi^{\prime \prime }}_{rc}\left(\xi \right){\psi^{\prime \prime }}_{sc}\left(\xi \right)d\xi K_{rsc}=\rho_{b}A_{b}\int_{0}^{H}\psi_{rc}\left(\xi \right)\psi_{sc}\left(\xi \right)d\xi +M_{k}\psi_{r}\left(H\right)\psi_{s}\left(H\right) \end{array}$$

Eqs. (11) and (12) can be expressed as quadratic forms of fiction:

(14) $$\begin{array}{ccc} E_{K,\max} & = & a^{T}K_{1}a+b^{T}K_{2}b+c^{T}K_{c}c\\ E_{Q,\max} & = & a^{T}M_{1}a+b^{T}M_{2}b+c^{T}M_{c}c \end{array}$$

Where

$$\begin{array}{ccc} & & a=\left[\begin{array}{c} a_{i}\\ a_{j} \end{array}\right],M_{n}=\left[\begin{array}{cc} M_{rrn} & M_{rsn}\\ M_{srn} & M_{ssn} \end{array}\right],K_{n}=\left[\begin{array}{cc} K_{rrn} & K_{rsn}\\ K_{srn} & K_{ssn} \end{array}\right]\\ & & *r,s=i.jn=1,2.c \end{array}$$

$a^{T}$ are the transposition of *a*.

Since elastic deformation does not consider energy loss, then the $\mu_{i}$ is given by:

(15) $$\mu_{i}^{2}=a^{T}K_{1}a+a^{T}K_{2}a+a^{T}K_{c}a/a^{T}M_{1}a+a^{T}M_{2}b+a^{T}M_{c}a$$

According to the principle of variational methods, the stationary value of a functional satisfies:

(16) $$\delta {\left(\mu_{i}\right)}^{2}=0$$

Whereδis the coefficient of the variable with minor changes in “*a”* according to the principle of variational methods.

Through deformation the following equation is obtained:

(17) $$\delta a^{T}\left[K_{1}+K_{2}+K_{c}-\mu_{i}^{2}\left(M_{1}+M_{2}+M_{c}\right)\right]a=0$$

From which

(18) $$\left[K_{1}+K_{2}+K_{c}-\mu_{i}^{2}\left(M_{1}+M_{2}+M_{c}\right)\right]a=0$$

Then, Set *a_i_=0, a_j_=1* and *a_j_=0, a_i_=1* respectively, and substitute into (18), finally subtract to obtain:

(19) $$\left(\mu_{i}^{2}-\mu_{j}^{2}\right)\left(M_{1}+M_{2}+M_{c}\right)=0$$

It follows that i≠j M is undoubtedly 0. Namely:

(20) $$\begin{array}{ccc} M_{1}+M_{2}+M_{c} & = & \rho_{b}A_{b}\left[\int_{-x_{0}}^{0}\psi_{i1}\left(x\right)\psi_{j1}\left(x\right)dx+\int_{0}^{x_{0}}\psi_{i2}\left(x\right)\psi_{j2}\left(x\right)dx\right]\\ & & +\;\rho_{c}A_{c}\int_{0}^{H}\rho_{c}A_{c}\psi_{ic}\left(\xi \right)\psi_{jc}\left(\xi \right)d\xi +M_{k}\psi_{ic}\left(H\right)\psi_{jc}\left(H\right)=0 \end{array}$$

And

(21) $$K_{1}+K_{2}+K_{3}=E_{b}I_{b}\left[\int_{-x_{0}}^{0}{\psi^{\prime \prime }}_{i1}\left(x\right){\psi^{\prime \prime }}_{J1}\left(x\right)dx+\int_{0}^{x_{0}}{\psi^{\prime \prime }}_{i2}\left(x\right){\psi^{\prime \prime }}_{j2}\left(x\right)dx+\int_{0}^{H}{\psi^{\prime \prime }}_{ic}\left(\xi \right){\psi^{\prime \prime }}_{jc}\left(\xi \right)d\xi \right]=0$$

It follows that when i=j,according to orthogonal normalization conditions:

$$M_{1}+M_{2}+M_{c}=\rho_{b}A_{b}\left[\int_{-x_{0}}^{0}\psi_{i1}^{2}\left(x\right)dx+\int_{0}^{x_{0}}\psi_{i2}^{2}\left(x\right)dx\right]+\rho_{c}A_{c}\int_{0}^{H}\rho_{c}A_{c}\psi_{ic}^{2}\left(\xi \right)d\xi +M_{k}\psi_{ic}^{2}\left(H\right)=1$$

$$K_{1}+K_{2}+K_{3}=E_{b}I_{b}\left[\int_{-x_{0}}^{0}{\psi^{\prime \prime }}_{i1}^{2}\left(x\right)dx+\int_{0}^{x_{0}}{\psi^{\prime \prime }}_{i2}^{2}\left(x\right)dx+\int_{0}^{H}{\psi^{\prime \prime }}_{ic}^{2}\left(\xi \right)d\xi \right]=\mu_{i}^{2}$$

Hence, the structure satisfies the orthogonality conditions:

(22) $$\begin{array}{ccc} M_{1}+M_{2}+M_{c} & = & \rho_{b}A_{b}\left[\int_{-x_{0}}^{0}\psi_{i1}^{2}\left(x\right)dx+\int_{0}^{x_{0}}\psi_{i2}^{2}\left(x\right)dx\right]+\rho_{c}A_{c}\int_{0}^{H}\rho_{c}A_{c}\psi_{ic}^{2}\left(\xi \right)d\xi +M_{k}\psi_{ic}^{2}\left(H\right)=\delta_{ij}\\ K_{1}+K_{2}+K_{3} & = & E_{b}I_{b}\left[\int_{-x_{0}}^{0}{\psi^{\prime \prime }}_{i1}^{2}\left(x\right)dx+\int_{0}^{x_{0}}{\psi^{\prime \prime }}_{i2}^{2}\left(x\right)dx+\int_{0}^{H}{\psi^{\prime \prime }}_{ic}^{2}\left(\xi \right)d\xi \right]=\mu_{i}^{2}\delta_{ij} \end{array}$$

Where $\delta_{ij}$ is Kronecker function, which meets the following condition:

(23) $$\delta_{ij}=\left\{ \begin{array}{c} 1\left(i=j\right)\\ 0(i\neq j) \end{array}\right.$$

### Derivation of modal functions

Firstly, the modal functions of the wave are derived as follows:

(24) $$\begin{array}{ccc} & & OA:\;\;\;\;a_{1}^{2}\psi_{i1}^{\left(4\right)}\left(x\right)+\mu_{i}^{2}\psi_{i1}\left(x\right)=0\\ & & OB:\;\;\;\;a_{1}^{2}\psi_{i2}^{\left(4\right)}\left(x\right)+\mu_{i}^{2}\psi_{i2}\left(x\right)=0\\ & & CD:\;\;\;\;{\bar{c}}_{1}^{2}\psi_{i}^{\left(4\right)}\left(\xi \right)+\mu_{i}^{2}\psi_{i}\left(\xi \right)=0 \end{array}$$

Subsequently , the general solution of horizontal bending moment wave mode functions of girders OA,OB and pier CD as motioned below:

(25) $$\begin{array}{ccc} & & \psi_{i1}\left(x\right)=C_{1}\sin K_{ib}x+C_{2}\cos K_{i4}x+C_{3}\sinh K_{ib}x+C_{4}\cosh K_{ib}x\\ & & \psi_{i2}\left(x\right)=D_{1}\sin K_{ib}x+D_{2}\cos K_{ib}x+D_{3}\sinh K_{ib}x+D_{4}\cosh K_{ib}x\\ & & \psi_{ic}\left(\xi \right)={\bar{E}}_{1}\sin K_{ic}\xi +{\bar{E}}_{2}\cos K_{ic}\xi +{\bar{E}}_{3}\sinh K_{ic}\xi +{\bar{E}}_{4}\cosh K_{ic}\xi \end{array}$$

Where$K_{ib}=\sqrt{\mu_{i}/a_{1}}$, $K_{ic}=\sqrt{\mu_{i}/{\bar{c}}_{1}}$are frequency relevant coefficients for bridge structures.

The wave mode function needs to comply with the following conditions:

The bending moment and boundary conditions are:

(26) $$\psi_{ib1}\left(-x_{0}\right)=\psi_{ib2}\left(x_{0}\right)=0,{\psi^{\prime \prime }}_{ib1}\left(-x_{0}\right)=0,\;\;{\psi^{\prime \prime }}_{ib2}\left(x_{0}\right)=0,$$

$$\psi_{ic}\left(0\right)=0,\psi {{}^{\prime }}_{ic}\left(0\right)=0$$

The force, support and continuity conditions are:

(27) $$\begin{array}{ccc} & & \psi_{ic}\left(H\right)=\psi_{ib1}\left(0\right)+\frac{E_{c}I_{c}{\psi^{\prime \prime \prime }}_{ic}\left(H\right)}{K_{v}},\;\\ & & E_{b}I_{b}\left({\psi^{\prime \prime \prime }}_{ib1}\left(0\right)-{\psi^{\prime \prime \prime }}_{ib2}\left(0\right)\right)=E_{c}I_{c}{\psi^{\prime \prime \prime }}_{ic}\left(H\right)=M_{k}{\psi^{\prime \prime }}_{ic}\left(H\right) \end{array}$$

Solving the Eq. (25) by introducing conditions (27), The general solution equation is:

(28) $$\begin{array}{ccc} & & \psi_{i1}\left(x\right)={\bar{E}}_{i1}\left[\frac{\sin K_{ib}\left(x+x_{0}\right)}{\cos K_{ib}x_{0}}-\frac{shK_{ib}\left(x+x_{0}\right)}{chK_{ib}x_{0}}\right]\\ & & \psi_{i2}\left(x\right)={\bar{E}}_{i1}\left[-\frac{\sin K_{ib}\left(x-x_{0}\right)}{\cos K_{ib}x_{0}}+\frac{shK_{ib}\left(x-x_{0}\right)}{chK_{ib}x_{0}}\right]\\ & & \psi_{ic}\left(\xi \right)=M_{2}{\bar{E}}_{i1}\left(\sin K_{ic}\xi -shK_{ic}\xi \right)+M_{3}{\bar{E}}_{i1}\left(\cos K_{ic}\xi -chK_{ic}\xi \right) \end{array}$$

By substituting $E_{b}I_{b}\left({\psi^{\prime \prime \prime }}_{ib1}\left(0\right)-{\psi^{\prime \prime \prime }}_{ib2}\left(0\right)\right)=E_{c}I_{c}{\psi^{\prime \prime \prime }}_{ic}\left(H\right)=M_{k}{\psi^{\prime \prime }}_{ic}\left(H\right)$,$\psi_{ic}\left(H\right)=\psi_{ib1}\left(0\right)+\frac{E_{c}I_{c}{\psi^{\prime \prime \prime }}_{ic}\left(H\right)}{K_{v}}$ , *M_2_, M_3,_* ${\bar{E}}_{i1}$ can be solved, respectively.

Additionally, $\mu_{i}$ can be derived by the orthogonality conditions (22),(23).

### Derivation of time function equations

Based on orthogonality condition(22), the time functions can be obtained as follows:

(29) $$T_{i}\left(t\right)+\mu_{i}^{2}{\ddot{T}}_{i}\left(t\right)=Z_{i}\left(t\right)$$

From which

(30) $${\ddot{Z}}_{i}\left(t\right)=-\left[\int_{-x_{0}}^{0}\rho A_{b}\frac{\partial^{2}X_{1g}\left(x,t\right)\psi_{i1}\left(x\right)}{\partial t^{2}}dx+\int_{0}^{x_{0}}\rho A_{b}\frac{\partial^{2}X_{2g}\left(x,t\right)\psi_{i2}\left(x\right)}{\partial t^{2}}dx+\int_{0}^{H}\rho A_{b}\frac{\partial^{2}X_{cg}\left(\xi ,t\right)\psi_{ic}\left(\xi \right)}{\partial t^{2}}d\xi \right]$$

Where

(31) $$S_{i}=\int_{-x_{0}}^{0}\rho_{b}A_{b}\psi_{i1}\left(x\right)dx+\int_{0}^{x_{0}}\rho_{b}A_{b}\psi_{i2}\left(x\right)dx+\int_{0}^{H}\rho_{c}A_{c}\psi_{ic}\left(\xi \right)d\xi$$

Since the initial velocity and displacement under horizontal excitation are assumed to be 0, hence:

(32) $$T_{i}\left(0\right)={\dot{T}}_{i}\left(0\right)=0$$

(33) $$T_{i}\left(t\right)=\frac{1}{\mu_{i}}\int_{0}^{t}\ddot{Z}\left(\tau \right)\sin\omega_{i}\left(t-\tau \right)d\tau =\frac{1}{\mu_{i}}S_{i}\int_{0}^{t}\ddot{D}\left(\tau \right)\sin\mu_{i}\left(t-\tau \right)d\tau$$

The dynamic displacement responses of the bridge structure under horizontal earthquake excitation can be expressed as follows:

(34) $$\begin{array}{ccc} & & X_{1d}\left(x,t\right)=-\sum_{i=1}^{\infty }\psi_{i1}\left(x\right)\cdot \left[\frac{1}{\mu_{i}}S_{i}\int_{0}^{t}\ddot{D}\left(\tau \right)\sin\mu_{i}\left(t-\tau \right)d\tau \right]\\ & & X_{2d}\left(x,t\right)=-\sum_{i=1}^{\infty }\psi_{i2}\left(x\right)\cdot \left[\frac{1}{\mu_{i}}S_{i}\int_{0}^{t}\ddot{D}\left(\tau \right)\sin\mu_{i}\left(t-\tau \right)d\tau \right]\\ & & X_{cd}\left(\xi ,t\right)=-\sum_{i=1}^{\infty }\psi_{ic}\left(\xi \right)\cdot \left[\frac{1}{\mu_{i}}S_{i}\int_{0}^{t}\ddot{D}\left(\tau \right)\sin\mu_{i}\left(t-\tau \right)d\tau \right] \end{array}$$

### Derivation of pounding response functions

At the beginning of the pounding process, the girder and shear key continue to vibrate with the frequency$\mu_{i}$,the initial pounding time is denoted as *t_1_*, define the initial position of the point O, the displacement and velocity conditions for pounding time *t_1_* should meet:

(35) $$X_{1b}\left(0,t_{1}\right)-X_{c}\left(H,t_{1}\right)\geq d\;\text{and}\;{\dot{X}}_{1b}\left(0,t_{1}\right)-{\dot{X}}_{c}\left(H,t_{1}\right)\geq 0$$

In which, *d* represents the initial gap between the girder and shear key, the positive direction is defined as moving towards the right. Once above conditions are met, it can be assumed that the shear key and the girder start to interact resulting in the outburst of contact forces. Conversely, if the above conditions are not activated, it is considered that the pounding has not been triggered.

Furthermore, from the perspective of pounding process, the total displacements are defined as the sum of the dynamic displacement response during separation and the instantaneous displacement of pounding process. The frequency variation caused by the pounding is minor and insignificant. The total displacements of girder and pier system in pounding process X_t,1b_, X_t,1c_ can be expressed as Eq. (36):

(36) $$\begin{array}{ccc} & & X_{t,1b}\left(x,t\right)=X_{1}\left(x,t\right)+X_{bF}\left(x,t\right)\\ & & X_{t,1c}\left(\xi ,t\right)=X_{c}\left(\xi ,t\right)+X_{cF}\left(\xi ,t\right) \end{array}$$

where$X_{g}\left(x\right)=X_{cg}\left(\xi \right)=D\left(t\right)$.

According to the Duhamel integral principle, the response of any excitation is equal to the convolution of its unit impulse response and the excitation. Thereby, the pounding deformation of the bridge structures, (assuming the pounding with the shear key on the right side, with the right side considered as the positive direction) is given by Eq. (37):

(37) $$\begin{array}{ccc} & & X_{bF}\left(x,t\right)=-\sum_{i=1}^{\infty }\psi_{ib1}\left(x\right)\int_{t_{1}}^{t}\psi_{i1}\left(x\right)F_{1}\left(t\right)\frac{1}{m_{i}\mu_{i}}\sin\mu_{i}\left(t-\tau \right)d\tau \\ & & X_{cF}\left(\xi ,t\right)=-\sum_{i=1}^{\infty }\psi_{ic}\left(\xi \right)\int_{t_{1}}^{t}\psi_{ic}\left(\xi \right)F_{1}\left(t\right)\frac{1}{m_{i}\mu_{i}}\sin\mu_{i}\left(t-\tau \right)d\tau \end{array}$$

Where *F_1_*(t) represents to the pounding force, and needs to match following equation:

(38) $$F_{1}\left(t\right)=\left[X_{t,1b}\left(0,t-t_{1}\right)-X_{t,1c}\left(H,t-t_{1}\right)\right]K_{v}$$

Where *m_i_* is the modal mass of the bridge structure system, and satisfy the Eq. (39):

(39) $$m_{i}=\int_{-x_{0}}^{0}\rho_{b}A_{b}\psi_{i1}^{2}\left(x\right)dx+\int_{0}^{x_{0}}\rho_{b}A_{b}\psi_{i2}^{2}\left(x\right)dx+\int_{0}^{H}\rho A_{c}\psi_{ic}^{2}\left(\xi \right)d\xi +M_{k}\psi_{ic}^{2}\left(\xi \right)$$

Eventually, substituting (36),(37), (39) into (38), the final equation for F(t) is as shown below:

(40) $$\begin{array}{ccc} & & \sum_{i=1}^{\infty }\psi_{i1}\left(0\right)\left[\begin{array}{c} T_{i}\left(t_{1}\right)\cos\mu_{i}\left(t-t_{1}\right)+\frac{1}{\mu_{i}}{\dot{T}}_{i}\left(t_{1}\right)\sin\mu_{i}\left(t-t_{1}\right)+\frac{1}{\mu_{i}}\int_{t_{1}}^{t}{\ddot{Z}}_{i}\left(\tau \right)\sin\mu_{i}\left(t-\tau \right)d\tau \\ -\frac{1}{m_{i}\mu_{i}}\psi_{i1}\left(0\right)\int_{t_{1}}^{t}F_{1}\left(\tau \right)\sin\mu_{i}\left(t-\tau \right)d\tau \end{array}\right]\\ & & \;-\;\sum_{i=1}^{\infty }\psi_{ic}\left(H\right)\left[\begin{array}{c} T_{i}\left(t_{1}\right)\cos\mu_{i}\left(t-t_{1}\right)+\frac{1}{\mu_{i}}{\dot{T}}_{i}\left(t_{1}\right)\sin\mu_{i}\left(t-t_{1}\right)+\frac{1}{\mu_{i}}\int_{t_{1}}^{t}{\ddot{Z}}_{i}\left(\tau \right)\sin\mu_{i}\left(t-\tau \right)d\tau \\ -\frac{1}{m_{i}\mu_{i}}\psi_{ic}\left(H\right)\int_{t_{1}}^{t}F_{1}\left(\tau \right)\sin\mu_{i}\left(t-\tau \right)d\tau \end{array}\right]\\ & & \;=\frac{F_{1}\left(\tau \right)}{K_{v}} \end{array}$$

**Fig. 6:**
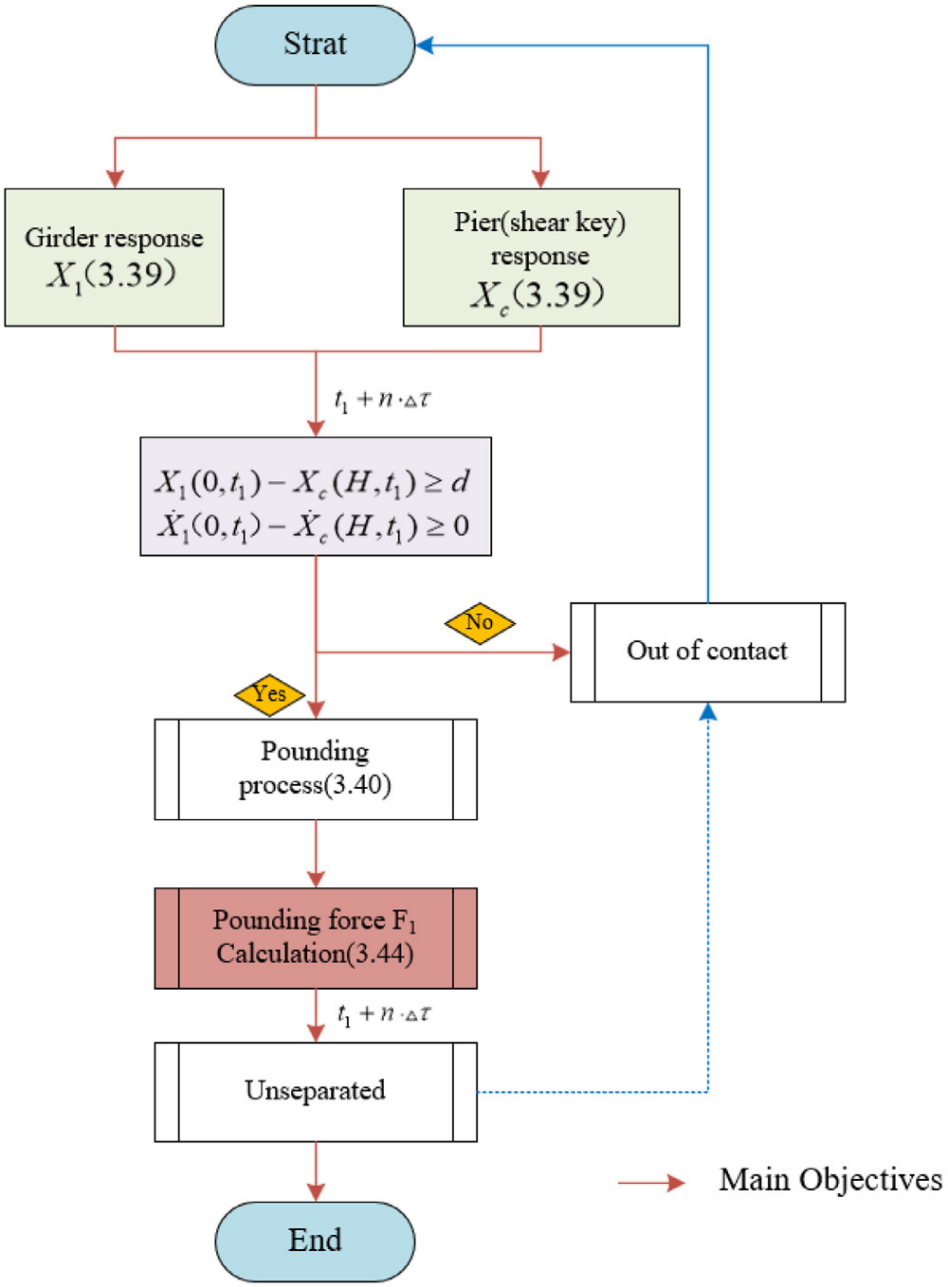
Criteria and calculation process for pounding.

According to the Eq. (40), it can be significantly observed that the displacement term contains an unknown variable related to the pounding force *F_1_(τ)*,τ represents the time step. Subsequently, the magnitude of the pounding force at each moment ($t_{1}+n\cdot ▵\tau$) can be conveniently solved through the spring compression length and the material stiffness. Procedure is as follows (Fig. 6):

## Summary

Based on a Beam-Spring-Beam + Concentrated Mass" continuum model, this paper is purposed primarily to expound a shear key pounding method for bridge structures. With a double-span continuous bridge as the research object, it will be analyzed.

Theoretically, the proposed model is developed to analyze the issue of pounding by deriving a series of structure response equations in the presence of horizontal seismic excitation. Meanwhile, by proving and inferring orthogonality conditions, the transient wave function expansion method and modal superposition method are developed for assistance in displacement response calculation.

The pounding process is modeled by developing the combined transient internal force method. Equivalently representing the pounding phase as a spring contact process, it is combined with the Duhamel integral method to express and solve the pounding/excitation responses. Through these methods in combination, the analytical expressions of motion of the system can be expended for extensive applications in the theoretical research on the dynamic response of systems under any conditions of loading.

## CRediT authorship contribution statement

**Chen Shutong:** Conceptualization, Methodology, Writing – original draft, Visualization. **Fadzli Mohamed Nazri:** Methodology, Writing – review & editing, Supervision. **An Wenjun:** Methodology, Writing – review & editing, Supervision. **Fu Hao:** Methodology, Writing – review & editing.

## Declaration of Competing Interest

The authors declare that they have no known competing financial interests or personal relationships that could have appeared to influence the work reported in this paper.

## Data Availability

No data was used for the research described in the article.
